# Rhino-Orbital-Cerebral Mucormycosis: A Rare Complication of Uncontrolled Diabetes

**DOI:** 10.1155/2022/6535588

**Published:** 2022-10-05

**Authors:** Rahaf F. Alanazi, Abdulrahman Almalki, Ali Alkhaibary, Fahd AlSufiani, Ahmed Aloraidi

**Affiliations:** ^1^College of Medicine, King Saud bin Abdulaziz University for Health Sciences, Riyadh, Saudi Arabia; ^2^King Abdullah International Medical Research Center, Riyadh, Saudi Arabia; ^3^Division of Neurosurgery, Department of Surgery, King Abdulaziz Medical City, Ministry of National Guard-Health Affairs, Riyadh, Saudi Arabia; ^4^Department of Pathology and Laboratory Medicine King Abdulaziz Medical City-CR, Ministry of National Guard Health Affairs, Riyadh, Saudi Arabia

## Abstract

**Introduction:**

Fungal infection of the central nervous system has become more common over the past two decades. It is frequently diagnosed in patients with underlying pathological conditions. We herein report a case of rhino-orbital-cerebral mucormycosis by outlining the clinical presentation, radiological images, histopathological findings, management plan, and its clinical outcome. *Case Description*. A 47-year-old man, known to have type 2 diabetes mellitus, presented with severe headache involving the left side of the face, numbness along the left V2 trigeminal nerve, ptosis and dryness of the left eye, short-term memory loss, and right hand numbness. He had a social history of being a bee farmer for which he was exposed to bee stings several times in the past. Neuroradiological imaging showed a left temporal ring-enhancing lesion, suggestive of abscess. The patient underwent craniotomy and resection of the lesion. The histopathological evaluation was suggestive of cerebral mucormycosis, fungal sinusitis, and invasive skull base mucormycosis.

**Conclusion:**

Rhino-orbital-cerebral mucormycosis is a fulminant fungal infection commonly diagnosed in patients with uncontrolled diabetes. Early diagnosis with radiological and histopathological evaluation is required to identify patients at risk of rhino-orbital-cerebral mucormycosis.

## 1. Introduction

Mucormycosis is a fungal infection with an estimated annual incidence of 1.7 per 1,000,000 inhabitants [1, 2]. The infection can be caused by inhalation of spores or damage to the skin/mucous membranes [2–4]. Immunosuppression, particularly in patients diagnosed with uncontrolled diabetes, is associated with a higher risk to develop mucormycosis [2, 5, 6]. It has been reported that the central nervous system is commonly involved in diabetic patients in which 33-49% of patients present with rhino-orbital-cerebral mucormycosis [1]. In the present article, we report a case of a 47-year-old male, with an uncontrolled diabetes, diagnosed with rhino-orbital-cerebral mucormycosis.

## 2. Case Description

### 2.1. History

A 47-year-old male presented to the emergency department complaining of severe left frontotemporal headache for three months. The patient reported numbness along the V2 trigeminal nerve of the left side and the right hand. It was associated with left face dryness, absent tears, ptosis of the left eye, and short-term memory loss. His past medical history was significant for diabetes mellitus type 2. There was no history of previous surgery to the skull base, trauma, use of immunosuppressive drugs, or seizures. His social history was significant for working in a bee farm since childhood. He reported that he used to use the bee stings as an alternative therapy, yet no similar presentation was reported before.

### 2.2. Examination

The patient was alert and oriented with a Glasgow Coma Scale (GCS) of 15/15. The pupils were 3 mm equal and reactive. There was left eye ptosis and decreased sensation to light touch on V2 trigeminal nerve distribution on the left side. The extraocular muscle movement was intact. The visual fields were intact to confrontation with no gaze preference. The patient looked dehydrated. The short-term memory was impaired. The muscle power was 5/5 all over the limbs.

### 2.3. Investigations

**Figure 1:**
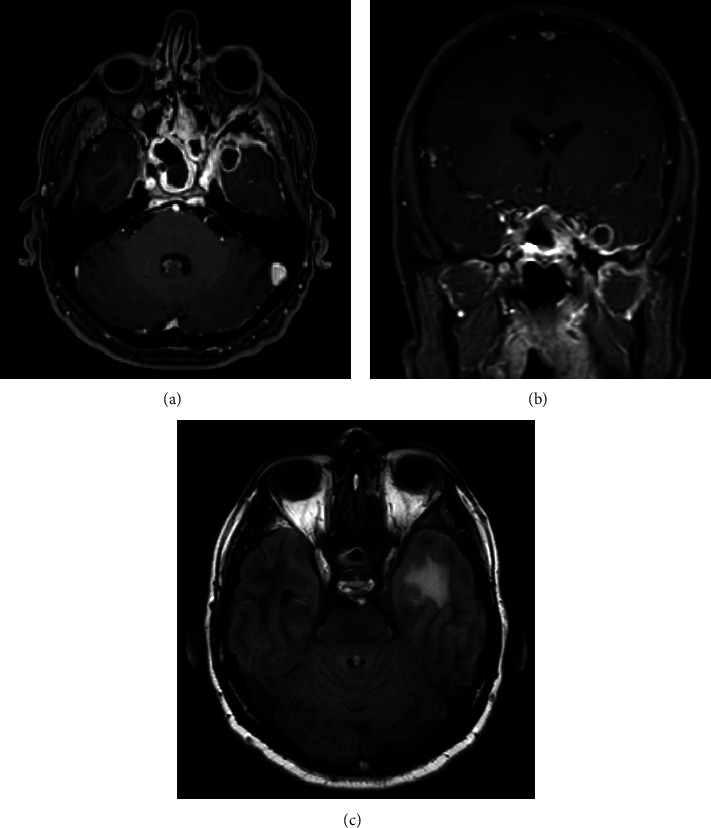
(a) Axial T1-weighted brain MRI with contrast. (b) Coronal T1-weighted brain MRI with contrast. (c) Axial T2-weight fluid-attenuated inversion recovery brain MRI. (a, b) The images demonstrate a left medial temporal lobe well-defined, ring-enhancing lesion measuring 1.2 × 1.3 × 1.2 cm in anteroposterior, transverse, and craniocaudal dimensions. There is bilateral pachymeningeal enhancement, mostly involving the middle cranial fossa, worse on the left side. Asymmetric enhancement of the left cavernous sinus is noted as well as left eye proptosis. There is enhancement of the left lateral recuts muscle. There are significant mucosal thickening, enhancement, and air-fluid level in the sphenoid sinus and ethmoid sinus. (c) A left temporal vasogenic edema is noted. (a–c) These findings are suggestive of invasive sinusitis complicated by skull base osteomyelitis with intraorbital and intracranial extension.

Laboratory investigations showed features of diabetic ketoacidosis. The random blood glucose level was 396 mg/dL. The anion gap was 18 mmol/L. The bicarbonate level was 21 mmol/L. The urine glucose was 1.000 mg/dL and ketones were 5 mg/dL. A brain magnetic resonance imaging (MRI) showed a left medial temporal ring-enhancing lesion with surrounding vasogenic edema, bilateral middle-cranial fossa pachymeningeal enhancement, and mucosal thickening of the sinuses (Figure 1).

### 2.4. Surgical Intervention

Considering the radiological findings, the patient underwent a left temporal craniotomy and resection of the brain abscess to establish the diagnosis. Evacuation of the pus was successfully performed. It was sent for frozen section, parament histopathology, and microscopic analysis of tissue cultures. The patient tolerated the surgery well with no complications.

### 2.5. Histopathological Evaluation

**Figure 2:**
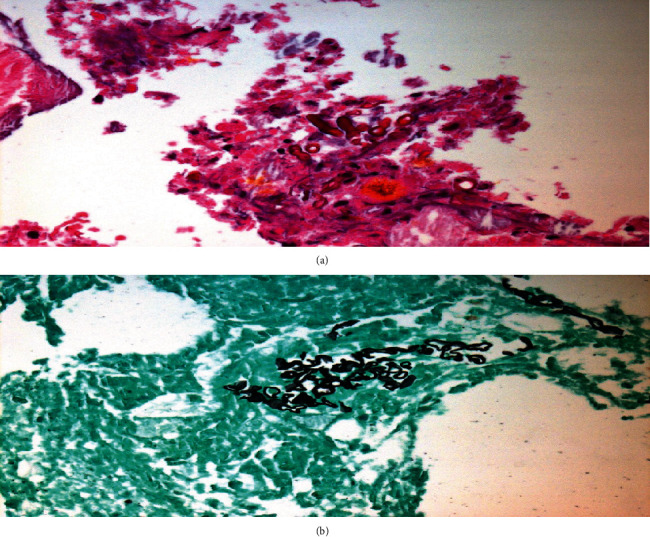
(a) Hematoxylin-eosin (H&E) stain. (b) Grocott Methenamine Silver (GMS) stain. (a, b) Examination of the left temporal lesions revealed an abscess due to a fungal infection (branching nonseptate hyphae). The morphology of these hyphae is suggestive of *Mucor* species.

The histopathological findings revealed an abscess due to a fungal infection suggestive of *Mucor* species (Figure 2).

### 2.6. Outcome and Follow-Up

Considering the radiological and histopathological findings, the patient was discharged in a stable condition with a long-term antifungal therapy for four months initially with regular radiological and clinical follow-up to assess the resolution of the infection and improvement of his clinical status.

**Figure 3:**
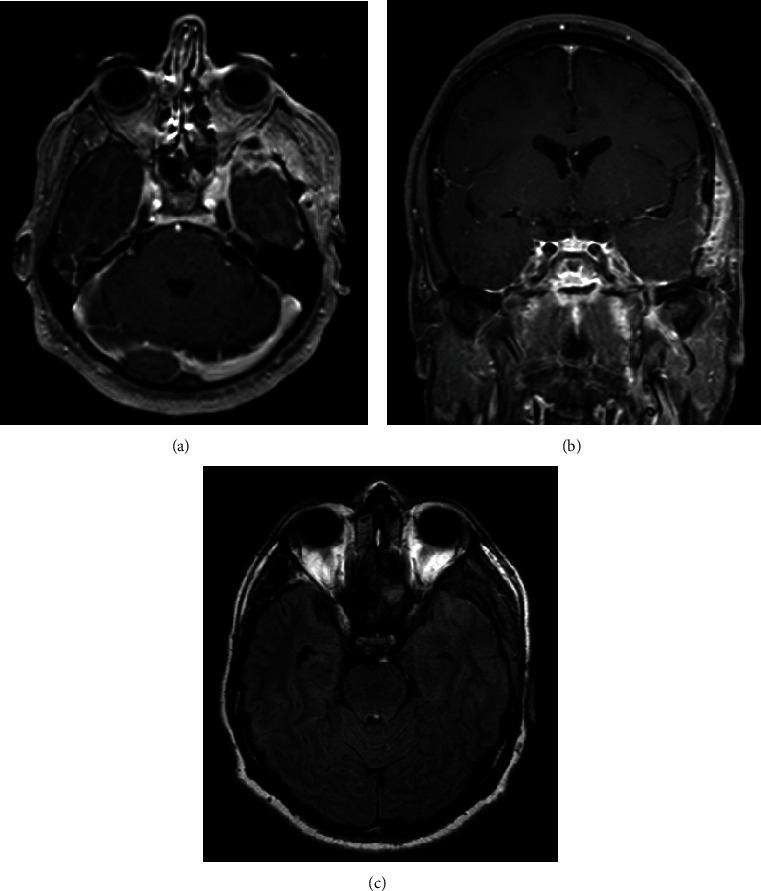
(a) Axial T1-weighted brain MRI with contrast. (b) Coronal T1-weighted brain MRI with contrast. (c) Axial T2-weight fluid-attenuated inversion recovery brain MRI. (a, b) Follow-up images after 9 months of resection demonstrating persistent pachymeningeal enhancement in the left temporal lobe with no evidence abscess formation. (c) There is resolution of the vasogenic edema in the left temporal lobe.

In follow-up after 9 months, the patient's left eye ptosis resolved, there is no evidence of memory disturbance no neurological deficits, and his diabetes is under control (Hgb A1c: 4.6%). A repeat brain MRI of abscess resection demonstrated no evidence of recurrence. However, there was evidence of pachymeningeal enhancement in the left temporal lobe, mostly representing persistent and chronic skull base osteomyelitis (Figure 3). Antifungal therapy was extended for another 7 months until there is complete neuroradiological resolution of enhancement.

## 3. Discussion

Mucormycetes are a group of opportunistic fungi typically diagnosed in patients with underlying pathologic conditions [1]. The disease commonly involves the central nervous system (CNS), affecting the rhino-orbital-cerebrum (33-49% cases), followed by cutaneous ([10-]16%), pulmonary ([10-]11%), disseminated (6%–12%), and gastrointestinal (2–11%) involvement [1].

Most of the rhino-orbital-cerebral involvement was reported in patients diagnosed with diabetes which evolved to become a predominant risk factor (58.9%–86.7%) and less commonly reported in hematological disorders, followed by renal failure or transplant recipients. Other forms such as cutaneous or soft tissue mucormycosis can primarily be diagnosed after skin disruption due to traumatic injuries, surgery, or burn.

The patient in the present article was diagnosed with diabetes for more than 10 years. He reported that he was compliant with his medications, and he denied any history of trauma, surgery, or burns. However, a risk factor was identified that might have contributed to his condition. He used to work as a bee farmer since childhood. In a study that examined the mycobiota and mycotoxins in bee pollen, Mucor species were the most common microscopic fungi isolated from bee pollen samples [7]. The infection is commonly caused by the inhalation of spores present in the air or by damage to the skin or mucous membranes.

Rhino-orbital-cerebral infection usually originates from the paranasal sinuses, with bone destruction and subsequent invasion of the orbit, eye, and brain. The clinical presentation of the disease may include headache, especially in the face, periorbital edema, often with loss of vision, fever, diplopia, rhinitis, and decreased mental function. Black nasal discharge (representing eschar), crusts, necrosis of the turbine, ulceration, and palatal perforation are also frequently observed.

The course of the disease is typically progressive over weeks to months. Diagnostic evaluation consisting of radiological findings of ring enhancement lesions with vasogenic edema can open the differential to intracranial fungal infection, including vascular infarct [8]. To narrow the differential diagnosis, radiological investigations should be accompanied with laboratory investigations and mycological/histological assessment. Biopsy is strongly recommended if mucormycosis is a potential diagnosis. Nuclear medicine has evolved into developing promising approaches in which the sensitivity of contrast media and radiotracers can be increased by engineering the composition of hyaluronic acid, a component of the extracellular matrix, and interactions with the living cells. [9]

Upon establishing the diagnosis of mucormycosis in a patient with malignancy, cranial, thoracic, and abdominal imaging studies are recommended to assess the extent of the disease. MRI or CT scan of the brain is strongly recommended for diabetic patients with facial pain, sinusitis, proptosis, ophthalmoplegia, or newly diagnosed amaurosis, to rule out sinusitis [10].

Treatment of rhino-orbital-cerebral mucormycosis relies on timely surgical intervention, antifungal treatment, and adequate control of predisposing factors. The combination of surgical debridement and antifungal agents results in lower mortality rates, as opposed to antifungal therapy alone. However, it is noted that surgical intervention can be less aggressive in patients with a slowly progressive disease [5, 6, 10].

The patient in the present article initially underwent craniotomy and resection of the brain abscess. After the cyst was opened and the pus was evacuated, a specimen was sent for pathology to determine the causative organism. Antifungal treatment was immediately initiated empirically to decrease the morbidity and mortality rates. Antifungal therapy was readjusted after the histopathological confirmation of the diagnosis.

It is estimated that the mortality rate varies between 30% and 97% depending on the time of diagnosis and progression of the lesion. However, survival is related to earlier diagnosis and application of multidisciplinary treatment approaches involving aggressive surgical debridement and antifungal therapy [1, 2, 6].

## 4. Conclusion

Rhino-orbital-cerebral mucormycosis is a fulminant fungal infection commonly present in patients with uncontrolled diabetes. Early diagnosis with neuroradiological and histopathological evaluation is required to identify patients at risk of mucormycosis. Recent technologies including the development of new approaches of nuclear medicine to detect the interactions between living cells, radiotracers, and contrast media can be of value to early and accurate diagnosis. Prompt surgical debridement, along with targeted antifungal therapy, is the optimal combination of choice for the management of such condition to halt the invasive progression of the disease.
